# The per-patient costs of HIV services in South Africa: Systematic review and application in the South African HIV Investment Case

**DOI:** 10.1371/journal.pone.0210497

**Published:** 2019-02-26

**Authors:** Gesine Meyer-Rath, Craig van Rensburg, Calvin Chiu, Rahma Leuner, Lise Jamieson, Steve Cohen

**Affiliations:** 1 Health Economics and Epidemiology Research Office (HE2RO), Department of Internal Medicine, Faculty of Health Sciences, University of the Witwatersrand, Johannesburg, South Africa; 2 Center for Global Health and Development, Boston University, Boston, Massachusetts, United States of America; 3 Strategic Development Consultants, Pietermaritzburg, South Africa; London School of Hygiene and Tropical Medicine, UNITED KINGDOM

## Abstract

**Background:**

In economic analyses of HIV interventions, South Africa is often used as a case in point, due to the availability of good epidemiological and programme data and the global relevance of its epidemic. Few analyses however use locally relevant cost data. We reviewed available cost data as part of the South African HIV Investment Case, a modelling exercise to inform the optimal use of financial resources for the country’s HIV programme.

**Methods:**

We systematically reviewed publication databases for published cost data covering a large range of HIV interventions and summarised relevant unit costs (cost per person receiving a service) for each. Where no data was found in the literature, we constructed unit costs either based on available information regarding ingredients and relevant public-sector prices, or based on expenditure records.

**Results:**

Only 42 (5%) of 1,047 records included in our full-text review reported primary cost data on HIV interventions in South Africa, with 71% of included papers covering ART. Other papers detailed the costs of HCT, MMC, palliative and inpatient care; no papers were found on the costs of PrEP, social and behaviour change communication, and PMTCT. The results informed unit costs for 5 of 11 intervention categories included in the Investment Case, with the remainder costed based on ingredients (35%) and expenditure data (10%).

**Conclusions:**

A large number of modelled economic analyses of HIV interventions in South Africa use as inputs the same, often outdated, cost analyses, without reference to additional literature review. More primary cost analyses of non-ART interventions are needed.

## Introduction

In economic analyses of HIV interventions, South Africa is often used as a case in point, due to both the size of its HIV-positive population and the number of people in need of and receiving services as well as the availability of good data on the course of its HIV epidemic and the outcomes of interventions. The fact that there is also often good local cost data available has however received less attention. As an example, a large number of economic evaluations of antiretroviral treatment (ART) options for low- and middle-income countries have used South Africa as a case study for decisions facing international donors supporting HIV programmes in sub-Saharan Africa more generally [1–7]. Very few of these analyses however use cost inputs from South Africa, despite the fact that the South African ART programme has been subjected to more cost analyses than any other ART programme outside the United States [8–25].

We present a summary of current unit costs for a large number of HIV services in South Africa that were generated during the analysis for the recent South African HIV and TB Investment Case [26]. The Investment Case used a novel optimisation methodology to inform local programme planners and both local and international funders about the most cost effective mix of interventions against both HIV and TB in South Africa over the next twenty years, using as the main outcome measure cost per life-year gained. It started with a long list of interventions proposed during a stakeholder workshop which was then subjected to a rigorous review of the evidence regarding each intervention’s effectiveness. Because the analysis optimised interventions and intervention coverage based on cost effectiveness, the cost of services was a central input into the analysis; at the same time, the analytical framework mandated that even though interventions could be excluded if there was no evidence as to their effectiveness, they could not be excluded if data on their cost was missing, leaving us with the task of establishing the cost of each of the interventions included in the Investment Case.

For this, we first conducted a systematic review of available published data on the costs of the selected interventions, based on primary cost analyses published between 2000 and 2016 that described a mode of delivery of the intervention that was relevant to South Africa, with or without a comparator population or intervention, and as part of any type of economic analysis, including cost and cost-effectiveness or cost-utility analyses. We then included into the Investment Case analysis those unit costs that were either, an update on such literature, or, if no literature was available, on data from recent expenditure analyses of relevant providers. Only in the absence of any such information did we use ingredient costing to establish the unit cost of a service, based on published data on the type and number of resources used in the intervention (such as staff, consumables, equipment, drugs and laboratory tests), and input costs (prices, salaries, etc) from a variety of sources. Across all types of unit cost, we used the same public-sector input costs to establish a common frame of reference.

## Methods

### Types of services included

The South African Investment Case followed the Investment Case framework used by UNAIDS to a certain extent [27] but additionally introduced the category of technical efficiency factor. The Investment Case framework includes biomedical interventions most often implemented by the healthcare sector alongside structural enablers (activities that have the potential to improve the efficiency of more than one intervention) and development synergies (investments into sectors other than health that have a positive effect on HIV outcomes amongst a broader range of impacts across different health and other development sectors).

This paper reviews available information on the costs for two types of services:

a) Interventions: These were all biomedical or behavioural interventions for which available evidence showed a direct impact on HIV risk, transmission, morbidity and mortality;

b) Technical efficiency (TE) factors: These were defined as activities that improve the technical efficiency of existing programmes, often by increasing their quality, uptake or coverage, but only affect a single intervention- in contrast to the enablers and synergies that have the potential to affect a number of interventions, possibly across different programme areas. (For a definition of “technical efficiency” and other economics terms used in this paper, please see Box 1.)

**Box 1. Definitions of economic terms.**

**Technical efficiency** in the context of the South African Investment Case refers to the maximisation of output (for example, HIV tests done) given a set level of inputs (for example, healthcare staff).

**Financial costs** only include accounting (or monetary) costs, whereas **economic costs** include both accounting costs and opportunity (or non-monetary) costs, for example volunteers’ time or donated goods.

**Micro-costing** is a method of cost estimation that enumerates and costs every input needed during a health intervention. Micro-costing may be used in either top-down or bottom-up costing, depending on the level of detail and precision required.

**Top-down costing** starts with the total expenditure on the intervention, and uses a metric (such as allocation factors based on patient volumes per service or similar) to assign total costs to individual services.

**Bottom-up costing** starts by identifying actual resource use (number of staff minutes, drugs etc. per patient year) of a sample of service recipients during the intervention, and multiplying the resource use by the cost of each resource from the same period as the resources were used. These resource costs are then summed to calculate the unit cost of the intervention.

**Ingredient-based costing** is used to cost interventions for which there is no data from economic study. It is similar to bottom-up costing but estimates resource use during the intervention based on assumptions, expert opinion, or other literature, rather than on a sample of service recipients, and applies costs taken from literature or other sources to reach a unit cost.

### Systematic review of cost data

We searched Pubmed for publications on the cost of each of the interventions, using a combination of MeSH and manually set search terms describing the intervention as well combinations of “economics”, “cost”, “cost analysis”, “costing”, “financial”, “budget” or “resource use”, at the level of title or abstract, as well as “South Africa” at the level of the text. We included papers published in any language between 01/01/2000 and 31/12/2016 (see S1 Table for the full list of search terms). In our full-text review we included papers containing primary cost data on at least one intervention with a mode of delivery of the intervention that was relevant to South Africa, and regardless of whether or not a comparator population or intervention had been included.

**Fig 1:**
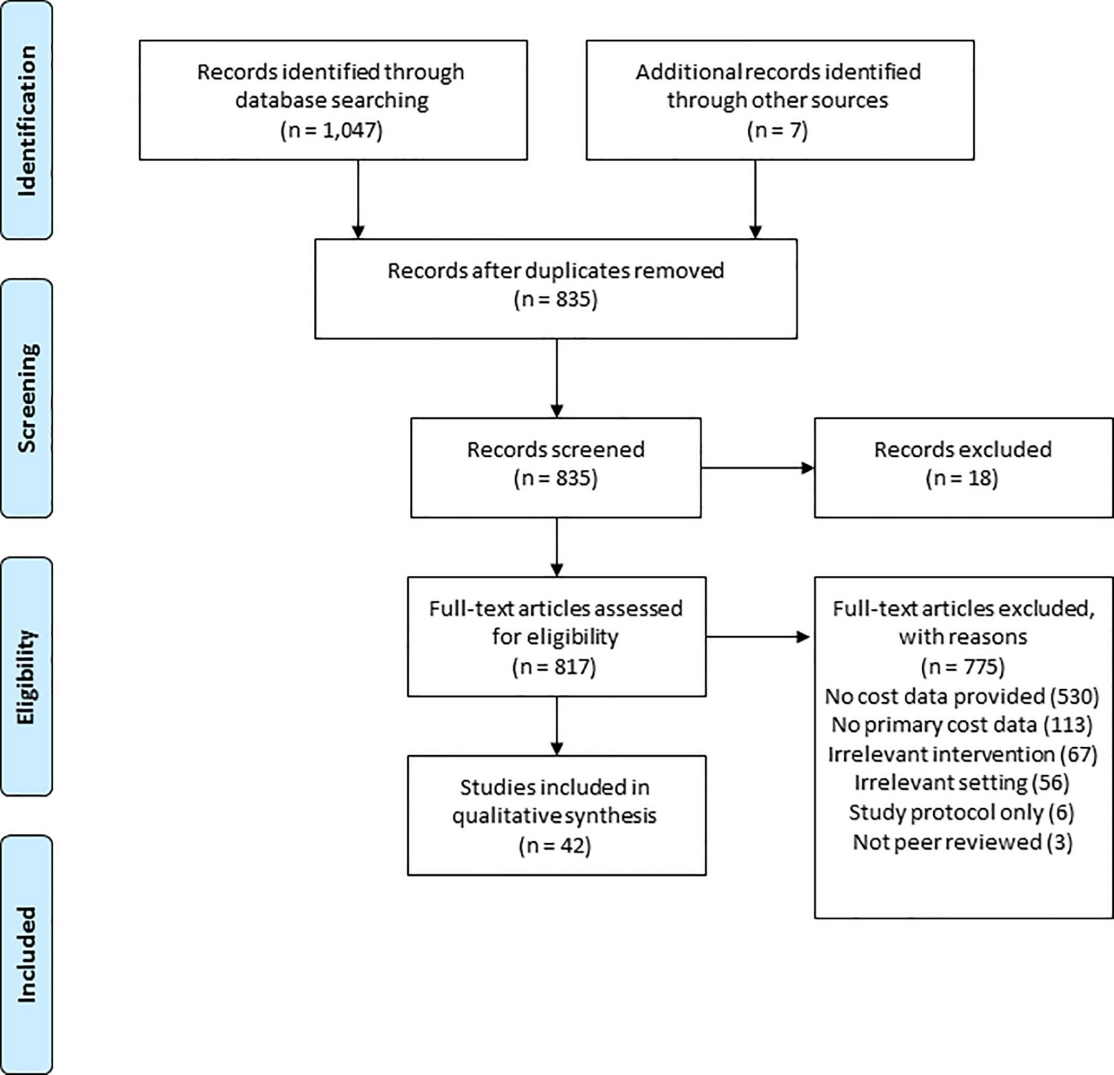
PRISMA diagram for systematic review.

We identified 1,047 papers through the search and another 7 references either mentioned in these papers or suggested by experts (Table 1). Of these 1,054 papers, 219 (21%) were duplicates, and 18 (2%) could not be found, leaving us with 817 papers for full-text screening (see Fig 1 for PRISMA diagram). Of these, 775 (95%) papers were excluded after full-text review, 530 (68% of those excluded) because no cost data was provided, 113 (15%) because no primary cost data was reported, ie, the cost data used in the paper came from another study, 67 (9%) because the intervention was not relevant to our search, 56 (7%) because the setting was not South Africa, and 6 (1%) because the paper described a study protocol only, not the results. Results of only 42 papers (5%) were included in the review.

**Table 1:** Results of literature review by intervention category.

Intervention category	Papers identified: Search	Papers identified: Other	Full text not found	Duplicates	Total assessed	Papers excluded							Included	% of all included papers
						Irrelevant intervention	Irrelevant setting	No cost data provided	No primary cost data	Study protocol only	Not peer reviewed	Total excluded		
Pre-ART care	10	0	1	0	9	1	1	4	2	0	0	8	1	2%
ART	469	3	8	54	410	35	23	255	62	3	2	380	30	71%
HCT	319	1	5	119	196	25	10	138	17	2	0	192	4	10%
PMTCT	4	0	0	4	0	0	0	0	0	0	0	0	0	-
MMC	49	1	0	6	44	0	5	16	18	1	1	41	3	7%
Condom distribution	65	0	1	9	55	2	5	46	2	0	0	55	0	-
PrEP	47	1	1	7	40	0	1	28	11	0	0	40	0	-
SBCC	27	0	0	5	22	1	5	16	0	0	0	22	0	-
PEP	6	0	1	0	5	0	0	5	0	0	0	5	0	-
Palliative care	8	0	0	1	7	0	0	6	0	0	0	6	1	2%
Inpatient care	43	1	1	14	29	3	6	16	1	0	0	26	3	7%
**Total**	**1,047**	**7**	**18**	**219**	**817**	**67**	**56**	**530**	**113**	**6**	**3**	**775**	**42**	100%

Data from these 42 papers were abstracted using an Excel sheet which was piloted on the first 10 papers, and updated thereafter. Data entries from one researcher were checked by another researcher working independently, and discrepancies resolved by reference to the original paper. The principal summary measures that we collected were the mean or median cost estimate with ranges, cost year, currency, and where relevant, exchange rate between the reported currency and USD in the cost year. Additional abstracted data included the year of publication and first author, the country or countries where cost data had been collected, the type of economic evaluation and its objective or objectives, whether the evaluation was part of a clinical trial, the target population and any population subgroups, a description of the intervention, intervention setting and level of care, the cost analysis method (economic vs. financial, and full vs. incremental cost), the cost data collection method and time period, whether costs were discounted and whether costs and any outcome indicators came from the same setting, whether a sensitivity analysis was done, and if so, of which parameters. Finally, we collected which cost categories had been reported as having been included in the cost analysis (costs of antiretroviral and any other drugs; laboratory, radiology, staff, consumable, overhead, capital, and transportation costs). S2 Table summarises the definition of each parameter collected from the included papers.

### Calculation of unit costs

For the purposes of this paper we define a unit cost as the cost per unit of output delivered by an intervention or technical efficiency factor (summarised as “services” in the following). The unit of output can be a person, test, visit, patient year, or a whole programme, but most often it will be a person reached by the particular intervention. We established the unit cost for each intervention using a number of methods:

First, we used data from papers identified in our review without further adjustment if the cost year was recent and the service described was represented implementation of the service under the most recent guidelines, for the target population in question and at the relevant level of care.

Second, wherever necessary we updated the published unit cost estimates to represent more recent input costs relevant to South Africa, such as staff salaries and drug costs, while maintaining information on the types and quantities of inputs required for the intervention. All cost data taken from published estimates was updated to 2016 South African Rand (ZAR) using the South African consumer-price index published by StatsSA [28] and relevant exchange rates if the estimate was not given in ZAR.

Third, where no unit cost could be found in the literature, cost was established using ingredient costing based on published data on the type and number of resources used in the intervention, and input costs from government commodity tenders, public servant remuneration documents, retail advertisements, government programme costings and budgets from South Africa’s current and past portfolio of grants with the Global Fund to Fight Aids, Tuberculosis and Malaria (GFATM). Finally, for selected interventions for which no published data on either unit cost or ingredients was available, we used information from budgets or expenditure records. This method was used for the cost of male medical circumcision and for the three social behaviour change communication campaigns included in the analysis.

The results of both the literature review and the cost calculation exercise are presented in both ZAR and US dollars (USD), using the 2017/18 period average exchange rate of 1 USD = 13.32 South African Rand.

### Structure of unit cost model

The analysis used a unit cost model that calculates the cost of each ingredient used in producing 1 output of an intervention and then aggregates the ingredient costs to arrive at a total unit cost per output of the intervention. For some ingredients assumptions regarding staff productivity are required to estimate the ingredient’s cost per output (for instance clients counselled per day by a counsellor). A useful benefit of building unit costs in this manner is that one is able to view the proportional contribution of each ingredient to the total unit cost.

For those interventions costed based on ingredients (rather than on literature or expenditure data), the cost per ingredient was the same across all interventions for which we used this ingredient- eg, for every intervention that required a primary healthcare nurse’s time, a minute of that nurse’s time costs the same. All ingredient costs were based on the most recently available public-sector data, either from sources in the public domain (such as the Department for Public Service Administration’s salary scales for salaries, the Essential Drug and ARV tender price lists list for drug prices and the price lists of the National Health Laboratory Service) or from budgets from past and current South African grants with the GFATM. Unit costs based on ingredients are denoted as “From ingredients*”* in Table 2; unit costs based on literature updated by more recent input prices are denoted as “From ingredients, based on Author (year)” in Table 2.

**Table 2:** Summary of results, methods and sources used in calculating unit costs. PC: Personal communication; “From ingredients”: For more details regarding the quantity and prices of ingredients, please see S4 Table.

Service	Unit cost [2017/18 ZAR]	Unit cost [2017/18 USD]	Cost value	Costing method/ source	Notes
1. Interventions					
ART (Adults)	R 3,318.62	$ 249.15	per patient year	National ART Cost Model (NACM) 201/18 [50]	Only relevant for 2017/18. Please contact the corresponding author for updates if required.
ART (Paediatric)	R 3,784.19	$ 284.10	per patient year	National ART Cost Model (NACM) 2017/18 [50]	
Male medical circumcision	R 1,770.29	$ 132.90	per circumcision	[51]	
Early infant male circumcision	R 885.14	$ 66.45	per circumcision	Based on above	Assumed to be 50% of adult MMC cost (D. Taljaard, PC)
Condom use	R 0.74	$ 0.06	per condom distributed	From ingredients	Weighted average of male and female condoms, including distribution costs
Male and female condom education	R 66.06	$ 4.96	per person trained	From ingredients, based on [52]	
PMTCT (mother not on any ART)	R 316.65	$ 23.77	per mother-baby pair	National ART Cost Model (NACM) [50]	
PMTCT B (mother not on lifelong ART)	R 2,125.74	$ 159.59	per mother-baby pair		
Infant testing at birth	R 442.23	$ 33.20	per test	From ingredients, based on [54]	
Infant testing at 6 weeks	R 416.59	$ 31.28	per test		
General population HCT (negative result)	R 48.24	$ 3.62	per test		
General population HCT (positive result)	R 74.83	$ 5.62	per test		
Testing of pregnant women (negative result)	R 104.43	$ 7.84	per test		
Testing of pregnant women (positive result)	R 111.64	$ 8.38	per test		
Testing of adolescents (negative result)	R 12.1	$ 0.91	per test		
Testing of adolescents (positive result)	R 6.19	$ 0.46	per test		
SBCC mass media campaign 1	R 3.39	$ 0.25	per person reached	Expenditure records from implementing agencies	Message: testing, multiple partners
SBCC mass media campaign 2	R 1,093.2	$ 82.07	per person reached		Message: condom usage and self-efficacy
SBCC mass media campaign 3			per person reached		Message: testing, condom usage and self-efficacy, MMC
Post-Exposure Prophylaxis (PEP)	R 1,918	$ 143.97	per patient	From ingredients	
Pre-Exposure Prophylaxis (PrEP)	R 1,647	$ 123.65	per patient year		
Young women, first year	R 1,900	$ 142.65			
Young women, every year thereafter	R 1,631	$ 122.48			
Young men, first year	R 1,915	$ 143.77			
Young men, every year thereafter	R 1,647	$ 123.65			
Female adolescents, first year	R 1,900	$ 142.64			
Female adolescents, every year thereafter	R 1,631	$ 122.42			
Male adolescents, first year	R 1,939	$ 145.55			
Male adolescents, every year thereafter	R 1,637	$ 122.91			
Female sex workers, first year	R 1,890	$ 141.86			
Female sex workers, every year thereafter	R 1,621	$ 121.68			
Men who have sex with men, first year	R 948.48	$ 71.21			
Men who have sex with men, every year thereafter					
Palliative care	R 1,694.49	$ 127.21	per patient	A. Lolliot, HPCA (PC)	
Inpatient care	R 989.47	$ 74.28			
pre-ART, <200 cells/microl	R 808.77	$ 60.72	per patient year	[53], adjusted to different CD4 strata	
pre-ART, 200–349 cells/microl	R 393.23	$ 29.52			
pre-ART, 350–500 cells/microl	R 2079.97	$ 156.15			
pre-ART, >500 cells/microl	R 1660.27	$ 124.64			
ART, <200 cells/microl	R 852.19	$ 63.98			
ART, 200–349 cells/microl	R 756.87	$ 56.82			
ART, 350–500 cells/microl	R 3,318.62	$ 249.15			
ART, >500 cells/microl	R 3,784.19	$ 284.10			
2. Technical efficiency factors					
Provider initiated counselling and testing (negative result)	R 56.07	$ 4.21	per test	Updated based on [54]	
Provider initiated counselling and testing (positive result)	R 82.66	$ 6.21	per test	Updated based on [54]	
Mobile HCT (negative result)	R 74.48	$ 5.59	per test	[34]	
Mobile HCT (positive result)	R 87.92	$ 6.60	per test		
Home based HCT (negative result)	R 69.73	$ 5.23	per test	[34]	
Home based HCT (positive result)	R 77.42	$ 5.81	per test		
Workplace HCT (negative result)	R 63.14	$ 4.74	per test	From ingredients	
HCT invitations to partners of pregnant women (negative result)	R 55.50	$ 4.17	per test	From ingredients	
HCT invitations to partners of pregnant women (positive result)	R 50.79	$ 3.81	per test	From ingredients	

Interventions for which the unit cost came directly from our literature review are denoted with the reference to the paper from which the cost was sourced in Table 2. For most of these estimates we updated input costs to more recent prices, using the same input costs as for those interventions costed based on ingredients. For those estimates that did not include enough details regarding ingredients and quantities, we forward-adjusted the estimate for inflation to 2016 costs as above. Estimates that were made available to us ahead of being published are denoted as “personal communication”, or “PC”, in Table 2.

Lastly, for those interventions for which neither literature on costs nor detailed ingredients nor expenditure data were available, most often because they were either so new that cost analyses had not yet been undertaken, we used expenditure data instead (denoted as “Expenditure records from implementing agencies” in Table 2).

It should be borne in mind that the purpose of this paper is to summrise potential sources of cost data rather than every sub-aspect of our own or other analysts’ cost analyses. For more information, we suggest to contact either the authors of the papers we reference or, if more information on the ingredients cost summarised in S4 Table is required, the corresponding author of this paper. Alternatively, we suggest searching the unit cost study repository of the Global Health Cost Consortium (https://ghcosting.org/pages/data/ucsr/app/index) which, at the time of writing, contained data from four of the papers included in our review).

## Results

### Literature review

Of the 42 papers identified through the literature review (5% of all papers included in full-text review), 30 (71%) reported on the cost of antiretroviral treatment in different models of care, 4 (10%) reported on HIV counselling and testing (HCT), 3 (7%) each on medical male circumcision and inpatient care, and 1 each (2%) on palliative care and pre-ART care (cotrimoxazole preventive therapy only) (Table 2). We did not find any cost papers reporting primary cost data relevant for South Africa on PMTCT, pre-exposure prophylaxis (PrEP), condom use, social and behaviour change communication (SBCC), or post-exposure prophylaxis (PEP). Papers found spanned the 12 years between 2005 and 2016, with slightly more papers (60%) published in the second six years than in the first.

Of the 42 papers, 34 (81%) reported resource use from the perspective of the healthcare provider (with the exception of one paper [29], the provider was the public sector), and 6 papers (14%) reported resource use from the patient perspective (with 5 and 1 papers, respectively, reporting on the costs associated with accessing ART and VMMC). Two papers (4%) reported on both provider and patient perspectives [30,31]. One paper (2%) on the cost of NIMART purported to have used a societal perspective by summing costs from the healthcare provider and patient perspectives [12]. Thirty-two papers (76%) presented full cost, 8 papers (19%) incremental costs, one paper (2%) reported both [32], and one paper (2%) did not give any information [33]. Thirty-two papers (76%) reported financial costs only, 5 papers (12%) economic costs, and another 5 papers (12%) both financial and economic costs. 55% of papers used a bottom-up approach to analysing resource use, 43% an ingredients or top-down approach, and one paper (2%) used both [34]. The majority of papers (62%) reported results as a point estimate only, without giving a range.

Cost categories included varied between intervention areas and perspectives. Cost items included in papers that only reported the provider perspective were staff costs (in 81% of these papers), consumables (69%), ARVs (in 61% of papers), non-ARV drugs (69%), laboratory tests (58%), equipment (58%), radiology (36%), inpatient cost (19%), transport costs (17%). Other specified cost items not attributable to these categories comprised nutritional support or supplements [35,36], communication costs [37], the costs of end of life care [34], field materials [38] and start-up costs [39], bank and interest charges [40] and insurance costs [41].

See S3 Table for the detailed cost results by paper.

### Unit costs

Table 2 gives an overview of the methods used in arriving at the unit cost of each intervention and TE factor included in the South African HIV Investment Case, the sources of all data, and the resulting unit cost. S4 Table furthermore gives details on the type, cost and quantities of ingredients included in each unit cost where applicable.

We were able to find estimates of the cost of 55% of the services in the literature, almost all of which we further updated to represent the most recent South African input prices. 35% of services were costed based on ingredients; the remaining 10% of services were costed based on expenditure data.

## Discussion

We reviewed the available literature on primary cost data for HIV services in South Africa. Despite HIV interventions making up a large proportion of the health interventions in South Africa for which cost estimates are available [42], and despite a large number of reviews showing that South Africa is home to more cost estimates for HIV interventions than any other low- and middle-income country [43–46], our search yielded limited results.

Only 5% of the papers on HIV interventions that used the term “cost” or “economics” as well as “South Africa” in the text reported the results of a primary cost analysis. Instead, a large number of modelled economic analyses of HIV interventions in South Africa which made reference to the same, often outdated, cost analyses, with no reference to any additional review of other literature. For example, a commonly cited cost source is one of the very early costs of ART in an NGO-run programme years before the public-sector roll-out [9]. This cost data was collected in 2003 and published in 2006. While the importance of the paper as one of very few sources of ART cost data in the early years of the public-sector ART programme cannot be overstated, it was still used as a cost source for papers published as recently as 2016 [47–49]- at which stage the data used in the models was over 10 years old.

We also found that the actual age of the input costs used in models was often lost through chains of referencing, where a modelled analysis used input costs that were the outputs of an older modelled analysis based on an even older primary analysis. In an effort to correct for outdated costs, those modelled analyses tended to inflate primary cost data over many years using general inflation indices such as the consumer price index, instead of sourcing and using more recent input prices for salaries, drug costs, etc, which skewed resulting estimates considerably, often upwards. Medical costs in South Africa have not increased with general inflation over the last 15 years. Labour costs, which are often the largest contributor to the cost of an intervention, have generally increased at a higher rate than inflation- for example, the introduction of Occupational Specific Dispensation in 2007 marked a structural shift in health worker remuneration. Similarly, drug costs, also a significant contributor to total costs, do not increase each year with inflation as the costs are negotiated through a tender process and may be fixed for a number of years, and in the past the prices tended to decrease as a result of tender negotiations. For these reasons we would advise caution in applying straight-line inflation adjustments to the costs of health interventions in South Africa over a number of years.

In line with the impact ART has on the total cost of the HIV programme in South Africa, more than two thirds of the identified 42 papers detailed the cost of ART. However, in our review we were able to find publications on the costs of a number of other interventions such as HCT, MMC, palliative and inpatient care. The majority of the reviewed publications included all cost categories relevant to the intervention under study, and more than half had collected data through bottom-up resource use analyses, often using large patient samples. However, only 62% of papers reported some estimate of uncertainty, increasing the risk of potential reporting bias.

Our review has a number of other limitations. Firstly, as mentioned above, we used inflation adjustment for the results of the literature review, in order to be able to present mean and median cost in a single cost year to aid comparison (see S3 Table). For the results of our own ingredient cost analysis, however, we added the details of the exact ingredients, quantities and unit prices included for each intervention and technical efficiency factor in S4 Table in order to allow other analysts to easily update our estimates to their settings and future prices. Secondly, our search strategy might have not retrieved all relevant papers, especially those reporting on results from smaller settings or using smaller sample sizes. Since we do not attempt to synthesize the evidence further, for example by creating a mean cost and cost range of a single intervention from several such reported estimates, we do not think that our analysis is subject to reporting bias.

In our review we were able to find literature detailing the results of primary cost analyses for five out of the 11 intervention categories included in the Investment Case, including for most of the HCT technical efficiency factors. The remaining interventions had to be costed using an ingredients-based approach, which is more prone to over- or underestimation than the detailed analysis of actual resources used in a bottom-up cost analysis. Only a small part of HIV services had to be costed based on expenditure. In this method, the relationship between inputs and outputs (in this case, services rendered) is even harder to establish, as invoices might have not been paid on time and the charges paid could have been higher than the actual cost of inputs. The quality of some of the results of this analysis is therefore limited; however, in each of these cases an estimate based on inferior methodology was deemed better than no estimate at all.

Our analysis yielded some insight into those interventions that need further cost analysis. In the area of HIV, more primary cost analyses are currently needed on the costs of PrEP, especially once routine delivery has started, social and behaviour change communication, and PMTCT. More work should also be directed to generate additional estimates of the cost of technical efficiency factors, including adherence interventions and different modalities of delivering HIV counselling and testing services, especially given the heightened demand for these factors.

## Supporting information

S1 Table Search strategies by intervention/ technical efficiency factor. (PDF)

S2 Table Definitions of variables collected in systematic review. (PDF)

S3 Table Results of literature review. (PDF)

S4 Table Details of ingredient-based unit costs [2017/18 ZAR]. (PDF)

S1 File PRISMA checklist. (PDF)
