# Supplementary material for: The per-patient costs of HIV services in South Africa: Systematic review and application in the South African HIV Investment Case
Source: PLoS One. 2019 Feb 26;14(2):e0210497. doi: 10.1371/journal.pone.0210497 (PMC6391029; doi:10.1371/journal.pone.0210497)
Supplement: S1 Table — (PDF) [file pone.0210497.s001.pdf]

Appendix S1: Search strategies by intervention/ technical efficiency factor

Appendix for the paper *"The per-patient costs of HIV services in South Africa: Systematic review and application in the South African HIV Investment Case"* by Gesine Meyer-Rath, Craig van Rensburg, Calvin Chiu, Rahma Leuner and Steve Cohen

**Intervention/ technical efficiency factor**	**Subject terms**
Cotrimoxazole	(cotrimoxazole[title/abstract] AND HIV[title/abstract])
ART	((Antiretrovirals*[title/abstract])) OR (ART[Title/Abstract])
NIMART	Nurse [title/abstract]) AND antiretroviral
Defaulter Tracer	((((((default[Title/Abstract]) OR defaulter[Title/Abstract]) AND HIV)) AND ((loss to follow up) OR (loss to follow-up) OR (LTFU) OR (loss to care) OR (retention))
SMS reminders	(((((short messaging service) AND HIV)) OR ((text message) AND HIV) OR (SMS AND HIV) OR ((text reminder) AND HIV)) OR ((Appointment reminder) AND HIV))
Home based ART	((((Home Based[Title/Abstract]) OR (Home treatment[Title/Abstract]) OR home[Title/Abstract] OR (Home-based[Title[Abstract])) AND HIV)
Adherence clubs	(((adherence clubs*) OR club*) AND HIV
Mobile HCT	((mobile[Title/Abstract] OR (community[Title/Abstract]) AND HIV) AND ((test* AND HIV)) OR HCT OR VCT)
Home-based CT (door-to-door)	((((Home Based[Title/Abstract]) OR (Home treatment[Title/Abstract]) OR home[Title/Abstract] OR ((Home-based[Title[Abstract])) AND HIV) OR ((Door to Door) AND HIV) AND (test*)
GP down referral	(((((GP) OR General Practitioner) AND (ART OR antiretroviral OR treatment)) AND HIV)
Community based adherence support for ART patients (adults)	("adherence support*" OR "treatment buddy" AND "community") AND (ART OR "antiretroviral" OR "treatment" OR "HIV")
MMC	(((Male circumcision[Title/Abstract]) OR MMC[Title/Abstract]) OR VMMC[Title/Abstract]) NOT Traditional[Title/Abstract]
Early infant male circumcision	((((Male circumcision[Title/Abstract]) AND infant) AND HIV)
Early infant male circumcision	(early infant male circumcision) OR EIMC
Condom use	((((Condom) AND (Uptake OR uptake OR demand OR distribution OR provision)) AND HIV)
Male and female condom education	condom* AND education AND HIV
Condom distribution in non-traditional outlets (hotels, shops, malls)	(Condom distribution) AND (alternate outlet) OR (distribution channel)
FSW: PrEP	((PrEP) or (pre-exposure prophylaxis) or (pre exposure prophylaxis)) and (sex work*)
Birth testing	(((Birth[Title/Abstract] OR infant[Title/Abstract] OR newborn[title/abstract])) AND (HIV AND TEST)) OR HCT) OR VCT)
Testing at 6 weeks	Infant HIV testing
CHWs to ensure reduced LTFU	((Community health worker*) and HIV) AND ((loss to follow up) OR retention)
HCT	(test* AND HIV) OR HCT OR VCT

Testing of pregnant women	(((((ANC OR antenatal OR PMTCT OR pregnant*) AND ((test* AND HIV[Title/Abstract) OR HCT[Title/Abstract] OR VCT[Title/Abstract])))
Testing of adolescents	((adolescent*[Title/Abstract]) OR (youth[Title/Abstract]) OR (young women[Title/Abstract]) OR (young men[Title/Abstract])) AND ((test* AND HIV[Title/abstract]) OR HCT[Title/abstract] OR VCT[Title/abstract])
PITC	((Provider initiated[Title/Abstract]) OR (provider-initiated[Title/Abstract]) OR PICT[Title/Abstract]) AND ((test*[Title/Abstract] AND HIV[Title/Abstract]) OR HCT[Title/Abstract] OR VCT[Title/Abstract])
Workplace HCT screening	(Workplace OR company) AND ((test* AND HIV) OR HCT OR VCT)
VCT Invitations	(Voluntary AND (HIV testing)) OR (Invitation AND (HIV testing))
SBCC Campaign 1	((SBCC OR (("social and behaviour change communication") OR ("social and behavior change communication") OR ("social behaviour change communication") OR ("social behavior change communication") OR ("behaviour change") OR ("behavior change") or ("mass media"))) AND "HIV"
Post-Exposure Prophylaxis (PEP)	((Post exposure prophylaxis) OR (post-exposure prophylaxis) OR PEP) AND HIV[Title/Abstract]
Pre-Exposure Prophylaxis (PrEP)	((Pre Exposure Prophylaxis) OR (Pre-Exposure Prophylaxis) OR PrEP) AND HIV[Title/Abstract]
PrEP for Adolescents	((Pre-Exposure Prophylaxis) OR (Pre exposure prophylaxis) OR PrEP) AND (adolescents OR youth OR (young women) OR (young men)) AND HIV
Microbicides	((microbicide*[Title/Abstract] OR gel[Title/Abstract] OR ring[Title/Abstract]) AND HIV[Title/abstract])
Palliative care	((Palliative care[MeSH Terms]) AND HIV) OR (Hospice AND HIV)
Inpatient care (pre-ART, <200 cells/microl)	(Inpatient care[Title/Abstract] OR hospital[Title/Abstract]) AND HIV
