# Supplementary material for: The per-patient costs of HIV services in South Africa: Systematic review and application in the South African HIV Investment Case
Source: PLoS One. 2019 Feb 26;14(2):e0210497. doi: 10.1371/journal.pone.0210497 (PMC6391029; doi:10.1371/journal.pone.0210497)
Supplement: S2 Table — (PDF) [file pone.0210497.s002.pdf]

Appendix S2: Variables collected

Appendix for the paper *"The per-patient costs of HIV services in South Africa: Systematic review and application in the South African HIV Investment Case"* by Gesine Meyer-Rath, Craig van Rensburg, Calvin Chiu, Rahma Leuner and Steve Cohen

**Variable name**	**Definition**
Year of publication	Year paper appeared in print. Not the same as the cost year. Only papers published after 2000 included.
Country/ countries	Only papers with data for South Africa were included.
Type of evaluation	Whether the study was a cost analysis, cost-effectiveness analysis, cost benefit analysis or cost minimisation, etc.
Intervention(s)	Definition of the intervention/s analysed in the paper, e.g. ART initiation, NIMART, adherence clubs.
Objectives	The main goal of the paper.
Part of clinical trial	As reported.
Target population	General population or key population or other sub-section of the general population.
Subgroups	Stratification of the target population, e.g. outcomes by CD4 count.
Setting	Urban or rural, single location or multiple locations.
Level of care	Primary health facility, secondary or tertiary.
Cost perspective	Provider or patient costs or both.
Costing method: Economic vs financial vs both	Economic cost includes costs for all resources used, including those which the provider may not have paid for. Financial costs include only costs which resulted in an actual outflow of cash.
Costing method	Full cost of the intervention estimated, or was only the incremental cost estimated.
Comparators	Different interventions, type of baseline.
Cost data collection method	Top down, bottom up or ingredients methods used.
Costing period (start - end)	Costing period is often different to the main study period if costing was not the main goal of the study.
Discount rate for cost	Rate used in the annualization of costs where needed.
Do resource use and costs come from the same setting?	Were all the costs collected from the same location the intervention was implemented, or were some taken from literature, or another location used as a proxy.
Sensitivity analysis	If done - univariate, multivariate or probabilistic.
If sensitivity analysis, which parameters?	List of parameters sensitivity analysis investigated.

Cost year (single year)	Year which costs are reported in.
Cost Currency	South African Rand (ZAR) or United States Dollar (USD).
Exchange rate (reported)	ZAR/Dollar rate, used to inflate costs to 2016 USD if needed.
Cost	In cost year and inflated to 2016 USD.
Cost per (time and/ or natural unit or outcome)	Cost per patient tested, cost per circumcision, etc.
Mean or median?	Which is reported in the paper. If the distribution of costs has a long tale the median will be lower than the mean.
Type of range	Standard deviation, 95% confidence interval, interquartile. Low range and high range captured.
Cost categories reported (ticked those that apply):	All drug
	ARVs
	Other drug
	Lab
	Imaging and radiology
	Staff
	Consumables
	Overhead
	Capital
	Transportation
	Other
