# Supplementary material for: The per-patient costs of HIV services in South Africa: Systematic review and application in the South African HIV Investment Case
Source: PLoS One. 2019 Feb 26;14(2):e0210497. doi: 10.1371/journal.pone.0210497 (PMC6391029; doi:10.1371/journal.pone.0210497)
Supplement: S3 Table — (PDF) [file pone.0210497.s003.pdf]

Appendix S3: Results of literature review

Appendix for the paper *"The per-patient costs of HIV services in South Africa: Systematic review and application in the South African HIV Investment Case"* by Gesine Meyer-Rath, Craig van Rensburg, Calvin Chiu, Rahma Leuner and Steve Cohen

No.	Author, year	Objective	Target population, setting, level of care	Cost perspective	Costing method	Costing period	Mean cost (2016 USD)	Range (2016 USD)	ARVs	Other drugs	Labs	Radiology	Staff/Consultation	Consumables	Overhead[1]	Equipment	Transport	Inpatient	Other
	**1. Cotrimoxazole**																		
1	Hausler (2006)	Cost and effectiveness of a package of TB and HIV interventions	General population in Cape Town; CHC, PHC and STI clinic	Provider	Full economic cost, ingredients approach	2001 - 2002	Screening for cotrimoxazole preventative therapy<br>$3.13 in community health centre<br>$1.57 primary health clinic<br><br>Cost of screening per person started on cotrimoxale preventative therapy<br>$7.84 in community health centres<br>$2.97 in primary health clinic<br><br>Cost of 6 months of cotrimoxazole preventative therapy<br>$12.54 community health centre<br>$9.40 primary health clinic	N/S		x			x	x	x	x			
	**2. ART outpatient cost**																		

[1] includes other capital costs in some instances

3	Chimbindi (2015)	Cost associated with accessing public sector care over different stages of illness (TB, pre-ART, ART)	General population in rural KwaZulu-Natal, PHC	Patient	Economic and financial costs, bottom up approach	2009-2010	Financial costs per month<br>$18.61 pre-ART<br>$17.84 on ART<br><br>Monetized time costs<br>$6.45 pre-ART<br>$9.49 on ART	All SD<br>$14.61-22.56<br>$15.37-20.38<br><br><br>$5.07-7.84<br>$4.11-14.88		x			x				x		Opportunity costs
6	Cleary (2012)	Explore barriers to access of ART at different treatment sites	Adult population (>18) in rural and urban treatment sites	Patient	Full financial costs, bottom up	N/S	Expenditure past month<br>$6.43- to reach ART facility (rural)<br>$18.34- all healthcare (rural)<br><br>$1.41- reach ART facility (Urban)<br>$5.77- all healthcare (Urban)	N/S		x							x		Patient health expenses
7	Cobb (2013)	Cost of applying WHO guidelines for the nutritional management of HIV-infected children	Children in a PHC in rural Hlabisa	Provider	Incremental financial costs, ingredients	2010	$56.25 /child nutrition, 26 weeks, requiring NCP-B only<br><br>$178.33 / child nutrition, 26 weeks requiring NCP-C and NCP-B<br><br>$923.63 average cost of supplying ART	N/S	x										Nutritional supplement
9	Deghaye (2006)	Provide comprehensive costing of HAART to health care workers in different hospitals	Health Care Workers	Provider	Full Economic and incremental financial costs, bottom up	2004	Total financial cost per year<br>$1379.18 urban<br>$896.74 peri-urban<br><br>Total economic cost per year<br>$1399.80 urban<br>$1066.89 peri-urban	N/S	x	x	x		x	x	x				

11	Harling (2007a)	Cost of a dedicated ART clinic over pre-ART and 2 years of ART	General population	Provider	Full financial costs, top down	March 2004-Feb 2006	Cost per patient month<br>$40.81 in 2004/05<br>$36.94 in 2005/06<br><br>Cost per visit<br>$55.49 in 2004/05<br>$42.16 in 2005/06	N/S	x	x				x	x	x			
16	Leisegang (2009)	Describe the direct costs and establish cost drivers over time in HIV managed care programme	19+ yrs old, ART naïve	Provider	Incremental financial costs, ingredients using AfA data base	N/S	Monthly cost<br><br>$96.96 before ART<br>$484.79 peri-ART<br>$193.92 on ART	N/S	x	x									
18	Long (2010)	Estimate the costs and outcomes of second line ART	Adult patients on second line treatment	Provider	Full financial costs, bottom up	N/S	Cost 12 months after initiating<br>$1045.39 all outcomes<br>$1278.26 care and responding<br>$1122.01 t in care and not responding<br>$482.88 no longer in care	N/S	x	x	x		x	x	x				
19	Martinson (2009)	Estimate the cost of initiating and maintaining patients on ART	Adults	Provider	Full financial costs, bottom up	October 2004-March 2005	Monthly costs<br>$96.48 overall per patient<br>$61.77 per patient not on ART<br>$117. per patient on ART<br>$105.24 for month prior to ART<br>$206.25 month of ART initiation<br>$127.69 after ART initiation		x	x	x	x	x			x			
21	Meyer-Rath (2013)	Annalise ART cost	Children (initiated ART, 13 yrs old)	Provider	Full financial costs, bottom up	2005-2009	Per 24 month outcome<br>$723.63 all in care<br>$652.11 all in sample<br>$304.88 not in care<br>$758.45 in care not responding<br>$707.63 in care responding		x	x	x	x	x	x	x				

22	Moshabela (2012)	Investigate factors associated with patterns of plural healthcare utilization	Adults	Patient	Incremental financial costs, bottom up	April 2008-March 2009	Report expenditure 4 weeks prior to ART visit<br>$5.65 private chemist<br>$8.47 self-care<br>$17.88 doctor<br>$6.59 primary health clinic	IQR<br><br>$2.82-12.23<br>$2.82-23.52<br>17.88-28.23<br>0.94-2.82 IQR		x			x						
25	Rosen (2007)	Estimate the costs patient occur in obtaining ART	Adults	Patient	Full financial and economic costs, bottom up	July 2005-June 2006	Total cost per visiit<br>$15.83 urban hospital<br>$10.20 informal settlement clinic<br>$18.88 rural clinic<br>$14.62 all						x				x		Opportunity cost
26	Rosen (2008)	Estimate outpatient cost per patient in care and responding to treatment	General population	Provider	Full financial costs, bottom up	N/S	Annual cost per patient initiated:<br>$747.59 referral hospital<br>$886.04 private GP<br>$921.64 HIV clinic<br>$1113.48 NGO clinic<br>$917.68 all sites<br><br>Cost to produce patient in care and responding:<br>$1115.46 referral hospital<br>$1703.84 private GP<br>$1463.54 HIV clinic<br>$1465.52 NGO clinic<br>$1422.01 all sites	N/A	x	x	x		x	x	x	x			
27	Tagar (2014)	Describe facility-level cost of ART in a random sample of facilities	General population	Provider	Full financial costs, top down	2011	Average cost of treatment per year<br>$521.46 weighted mean<br>$597.71 simple mean<br><br>Average 1st line cost<br>$160 cost for adults		x	x	x		x	x	x	x			Nutritional

28	Vella (2011)	Identify which delivery profiles of ART sites influenced retention and at what cost patients were retained	General population	Provider	Full financial costs, bottom up	April 2005-March 2006	6 month cycle cost:<br><br>$471.58 Part time MD part time nurse, <200 new patients per MD per yr<br>$484.38 Part time MD part time nurse, >= 200 new patients per MD per yr<br>$509.98 full time MD + full time nurse, <200 new patients per MD per yr<br>$531.64 full time MD + full time nurse, >= 200 new patients per MD per yr<br><br>Incremental cost per patient on treatment after 10 years:<br><br>$8864.60 Part time MD part time nurse, <200 new patients per MD per yr<br>$12081.02 full time MD + full time nurse, <200 new patients per MD per yr	6 month cycle (95%)<br>$447.96-495.21<br><br>$476.51-492.26<br><br>$502.10-517.86<br><br>$506.04-557.24<br><br>ICERs (95%)<br><br>$8,369-9,367<br><br>$10,430.97-13,709	x	x	x	x	x	x	x	x			
29	Wouters (2010)	Investigate characteristics of patients enrolled in public sector ART programme in Free State	General population	Patient	Full financial costs, bottom up	2004-2006	$1.22 mean transport cost	SD<br>$0-2.31									x		
	**2. ART in- and outpatient cost**																		
5	Cleary (2006)	Cost and cost effectiveness of HIV services, cost per LY and QALY gained	General population in Khayelitsha, Cape Town, HIV clinic for ART, tertiary and secondary facility for TB	Provider	Full economic cost, ingredients and step down approach	N/S	Cost per clinic visit<br>$22.11 ART<br>$21.64 no-ART<br><br>Incremental cost effectiveness<br>$1,187.25 per LY gained<br><br>$1,329.62 per QALY	$984-1,187 per LY gained<br>$1,102-1,330 per QALY	x	x	x	x	x	x	x	x		x	

12	Harling (2007b)	Analyse ART costs over 3 treatment periods	General population	Provider	Full financial costs, top down	March 2004-Feb 2006	Pre ART costs<br>$409.19 intention to treat<br>$380.45 on program<br><br>Treatment costs per patient year 1<br>$2,533.71 intention to treat<br>$2045.73 on program<br><br>Treatment costs per patient year 2<br>$1,389.83 intention to treat<br>$1.392.74 on program	N/S	x	x	x			x	x	x		x	
15	Kevany (2009)	Determine the costs of inpatient and outpatient at a dedicated ARV referral unit	Older than 13 yrs	Provider	Incremental economic costs, bottom up	Apr & May 2005	Outpatient costs<br><br>$194.87 cost per patient<br>$148.47 cost per visit<br><br>Inpatient costs<br>$148 cost per inpatient<br>$488.49 cost per day	N/S	x	x	x	x	x					x	
20	Meyer-Rath (2012)	Compare resource use and cost between 2 settings	General population, CD4 < 200	Provider	Full financial costs, bottom up	April 2006-Dec 2008	Inpatient costs per year<br>$304.99 urban<br>$366.34 semi-urban<br><br>Outpatient costs per year<br>$606.48 urban<br>$610.86 semi-urban<br><br>Total costs per year<br>$911.47 urban<br>$977.20 semi-urban	95% CI<br>$127.08-482.03<br>$70.99-662.57<br><br><br>$552.14-660.81<br>$589.82-631.01<br><br><br>$701.13-1121.80<br>$680.09-1273.42	x	x	x	x	x	x	x	x		x	

23	Nachega (2010)	Determine the effect of ART adherence on the direct health care costs among adults	Adults	Provider (private)	Incremental financial costs, top down	N/S	Mean monthly cost<br>$356.45 all patients<br>$362.14 adherence 4-45%<br>$402.15 adherence 40-75%<br>$358.86 adherence 75-91%<br>$302.26 adherence 92-100%<br><br>Median monthly cost<br>$203.44 all patients<br>$202.47 adherence 4-45%<br>$170.66 adherence 40-75%<br>$208.26 adherence 75-91%<br>$215.97 adherence 92-100%	SD<br>$0-996.45<br>$214.52-938.80<br>$400.61-1,204.90<br>$134.31-852.02<br>$0- 935.90<br><br>IQR<br>$147.52-303.71<br>$135.95-323.96<br>$102.20-319.13<br>$140.77-345.17<br>$151.37-327.81	x	x	x	x	x					x	Other
24	Naidoo (2015)	Measure the effectiveness of initiating ART at different points in TB treatment	General population	Provider	Incremental financial costs, bottom up	2005-2010	Total median variable cost per patient month<br><br>$100.57 with ART within 4 weeks<br>$96.96 ART after intensive phase of treatment<br>$86.59 ART after completing treatment	IQR<br><br><br>$93.38-142.62<br>$89.99-126.49<br><br>$78.10-113.76	x	x	x	x	x	x				x	
	**3. Inpatient cost**																			

8	Smith de Cerif (2009)	Determine whether reasons for hospitalization were different for HAART vs non-HAART	Adult population (>18), secondary hospital, Cape Town	Provider	Full financial costs, ingredients (some costs from Cleary 2006)	July 2003-March 2004	Total hospitalization cost<br>$1513.91 in HAART group<br>$1401.09 in NON-HAART group	IQR<br>$777.91-2855.9<br>$873.53-2231.64	x	x	x	x	x	x	x	x			
42	Meyer-Rath (2013)	Compare hospitilisation rates and costs in cohort of HIV+ patients before and after ART initiation	Adult population (over 18 years)	Provider	Full financial costs, ingredients approach	2009	Cost per patient per year, pre ART<br>$142.21 at 100 cells/mm³<br>$74.34 at 101 – 200 cells/mm³<br>$56.56 at 201 – 350 cells/mm³<br>$35.55 at >350 cells/mm³<br><br>Cost per patient per year, on ART<br>$330.49 at 100 cells/mm³<br>$74.34 at 101 – 200 cells/mm³<br>$56.56 at 201 – 350 cells/mm³<br>$35.55 at >350 cells/mm³	95% CI<br>$76.76-234.33<br>$39.59-122.82<br>$56.56-83.23<br>$20.20-54.14<br><br>$120.40-694.91<br>$75.96-327.25<br>$58.18-139.79<br>$18.58-64.64											

43	Sinha (2012)	Determine the economic burden of acute lower respiratory tract infection (ALRTI) in South Africa	HIV+ and HIV - Children (>6 months)	Provider and patient	Full financial costs, top down for bed days, bottom up for other resource use	2000 – 2001	HIV+ costs, cost per ALRTI episode<br>$969.61, direct paediatric ward costs, (retrospective period)<br>$1,117.10 direct paediatric ward cost (prospective period)<br>$198.28 short stay ward costs (prospective period)<br>$11.88 patient costs<br><br>HIV- costs, cost per ALRTI episode<br>$804.31, direct paediatric ward costs, (retrospective period)<br>$809.10.10 direct paediatric ward cost (prospective period)<br>$193.90 short stay ward costs (prospective period)<br>$10.37 patient costs	95% CI<br>$4874.53-1,064.44<br>$892.08-1342.11<br><br>$184.32-193.90<br><br>$8.78-16.76<br><br><br>$711.75-896.07<br><br>$646.32-971.88<br><br>$184.32-212.25<br><br>$8.78-16.79		x	x	x	x			x	x		Opportunity cost
48	Long (2016)	Report the primary reasons for HIV-related admissions at a regional, urban hospital	Adult (>18)	Provider	Full financial costs, bottom up	2012-2013	Cost per HIV+ admission<br>$3,518 renal disease<br>$2,298 drug adverse event<br>$2,205 endocrine and metabolic disease<br>$2,172 HIV and related OI<br>$2,135 gastro-intestinal disease<br>$2,096 unknown reason for admission<br>$1,818 hematologic disorder<br>$1.719 parasitic infection<br>$1,714 TB and other mycobacteria<br>$1,677 musculo-skeletal<br>$1,250 other<br>$1,590 all	N/S		x	x	x		x	x	x	x		
	**4. NIMART**																		

30	Barton (2013)	Estimate cost effectiveness of nurse-led ART vs doctor lead ART	Adult population (16+)	Provider	Full financial costs, bottom up	2008-2010	Cost at 12 months post enrollment, patients CD4 <=350 and not yet on ART<br><br>$383.05 nurse lead ART<br>$285.70 doctor lead ART<br>$23,444 per death averted<br><br>Costs at 12 months post enrollment, Patients on ART at enrollment<br>460.68 nurse lead ART<br>402.91 doctor lead ART<br>12,042 per undetectable viral load	95% CI $17,529-19,625<br><br>$7,079-9,550	x	x			x	x	x			x	Set up costs
31	Foster (2012)	Evaluate indirectly supervised pharmacists' assistant (ISPA) and nurse based care against full time pharmacist	General population	Societal	Incremental financial and economic costs, bottom up	2009	Average annual costs:<br>$109.67 per patient with full time pharmacist (baseline)<br>$10.68 incremental cost for patient through ISPA<br>$27.11 incremental cost per patient through nurse<br><br>Average societal cost per visit:<br>$14.10 with full time pharmacist (baseline)<br>-$2.89 incremental cost with ISPA<br>-$0.11 incremental cost with nurse<br><br>Average cost to patient per visit<br>$8.54 with full time pharmacist (baseline)<br>-$2.21 incremental cost with ISPA<br>-$3.19 incremental cost with nurse<br><br>Average cost to patient per visit<br>$5.57 with full time pharmacist (baseline)<br>-$0.69 incremental cost with ISPA<br>$3.07 incremental cost with nurse	N/S					x			x	x		Indirect patient costs

32	Long (2011)	Evaluate cost effectiveness of down referring from doctor managed to nurse managed ART in primary health care	Stable patients on ART > 11 months	Provider	Full financial costs, bottom up	N/S	Cost per patient:<br><br>514.55 DR/Hospital<br><br>463.95 Down referral<br><br><br>Cost per patient in care and responding:<br>526.01 DR/Hospital<br>469.98 Down referral	Cost per patient (SD)<br>$379.95-649.15<br><br>$70.40-557.51<br><br><br>Cost in care (SD)<br>$403.81-648.20<br>$385.67-553.69	x	x	x		x	x	x	x			
45	Fatti (2016)	Compare pharmaceutical care quality, clinical outcomes and provider staff costs between task shifting pharmaceutical care models	ART naïve adults, 16 yrs and older	Provider	Incremental financial costs, bottom up	Jan 2013-Dec 2013	Mean staff cost per visit<br>$130 through ISPA<br>1.82 through nurse managed<br><br>Staff cost per item dispensed<br>$0.41 through ISPA<br>$0.81 through nurse managed	N/S					x						

53	Long (2016)	Compare outcomes and costs of NIMART between primary health clinics and hospital based HIV clinics	Adults >18yr	Provider	Full financial costs, bottom up	2014	Average cost for all patients:<br>323.74 HIV outpatient<br>201.63 PHC<br><br>Average cost per patient alive & in care<br>405.15 HIV outpatient<br>225.29 PHC<br><br>Average cost per dead/LTFU<br>115.49 HIV outpatient<br>94.66 PHC	All patients (95%)<br>308.60-338.89<br>193.11-210.15<br><br>Alive & in care<br>399.47-427.87<br>220.56-230.97<br><br>Dead/LTFU<br>100.34-129.69<br>82.36-106.97	x	x	x	x	x		x	x			
	**4. SMS notification**																		
33	De Tolly (2012)	Investigate effectiveness of SMS to encourage HCT	General population	Provider	Full financial costs, bottom up	N/S (?2011)	$0.02 per sms<br><br>$1.76 per additional tester	N/S											Cost per sms
	**5. Adherence clubs**																		

34	Bango (2016)	Assess cost effectiveness of lay health worker led group adherence clubs and describe and evaluate the associated patient costs	Adult, stable patients	Provider and patient costs	Full financial costs, top down	2011	Total cost per visit:<br>$12.97 adherence club<br>$14.16 standard of care<br><br>Patient transport costs<br>$0.71 adherence club<br>$1.15 standard of care<br><br>Total cost per patient yr<br>$235.45 adherence club<br>$293.53 standard of care	N/S	x		x		x	x	x	x	x		Patient opportunity costs
	**6. HIV counselling and testing (HCT)/ Voluntary counselling and testing (VCT)**																		
36	Basset (2007)	Evaluate the yield of routine VCT vs provider referred VCT	General population	Provider	Full financial costs, top down	Jan – Mar 2005	Cost per test<br>$9 referral<br>$8.56 routine VCT<br><br>Cost per HIV positive person identified<br>$13.48 referral<br>$25.81 routine VCT	N/S					x	x	x				
1	Hausler (2006)	Cost and effectiveness of a package of TB and HIV interventions	General population in Cape Town; CHC, PHC and STI clinic	Provider	Full economic cost, ingredients approach	2001 - 2002	Cost per VCT completed<br>$14.10 at community care center<br>$17.24 at primary health clinic<br>$10.97 at STI clinic<br><br>Cost per HIV infection averted<br>$145.74 at community health center<br>$175.51 at primary health clinic<br>$104.99 at STI clinic	N/S		x			x	x	x	x			

37	McConnel (2005)	Determine the cost of a client completing VCT through rapid test technology	General population	Provider	Full economic and financial costs, top down	June 2002 – May 2003	Cost per counselling and test<br>$116.45 economic cost<br>$68.85 financial cost<br><br>Cost per completed VCT sequence<br>$121.89 economic cost<br>$72.07 financial cost	N/S				x	x	x	x				
	**7. Home HIV counselling and testing**																		
39	Smith (2015)	Evaluate impact and cost effectiveness of a community based home HTC package	Adult population (over 18 years)	Provider	Full financial costs, bottom up and ingredients	N/S	Cost per HIV negative test<br>$20.52 research model<br>$8.67 operation model<br><br>Cost per HIV positive test<br>$35.29 research model<br>$23.28 operational model<br><br>Incremental cost effectiveness<br>$21,920 per HIV infection averted<br>$1,335 per DALY averted	90% of model variability<br>$11,300-<br>$75,900<br>$1080-1760	x		x		x	x	x	x	x		End of life care
40	Tabana (2015)	Examine the cost effectiveness of home based testing in rural SA	General population	Provider	Full financial costs, ingredients approach	Jan – Dec 2010	$23.08 cost per home based client<br>$30.24 cost per clinic client	N/S					x	x	x	x			Field materials
	**8. Palliative care**																		

41	Hongoro (2011)	Establish cost and cost drivers of a hospital palliative outreach service	General population	Provider	Full financial costs, top down	2007/ 2008	Cost per home visit<br>$77.51 not including capital costs<br>$109.87 including capital costs<br><br>Cost per home visit registered patient<br>$724.00 not including capital costs<br>$1,026.29 including capital costs<br><br>Cost per hospital visit<br>$87.45 not including capital costs<br>$119.82 including capital costs<br><br>Cost per hospital registered patient<br>$156.88 not including capital costs<br>$214.94 including capital cost	N/S		x			x	x	x		x		Bank and interest charges
	**9. Point of care CD4 testing**																		
44	Larson (2012)	Estimate cost of CD4 point of care testing	N/A	Provider	Full financial costs, bottom up	2010	$19.42 cost per test<br><br>$20.02 cost per successfully completed test	N/S					x	x		x			Insurance cost
	**10. Voluntary medical male circumcision (VMMC)**																		

46	Tchuenche (2016a)	Derive the unit cost of delivering VMMC at the facility level and to identify the level of spending currently incurred for VMMC demand creation	Men and boys	Provider	Full financial costs, ingredients	Jan-Dec 2014	Per circumcision performed<br>$124.95 across all facilities<br>$131.11 in facilities with outreach services<br>$123.15 in facilities without outreach services<br>$110.75 urban facilities<br>$137.26 peri-urban facilities<br>$111.70 rural facilities<br>$145.78 in hospitals<br>$114.54 health care centre/clinic	SD<br>$116.24-145.97<br>$115.37-130.94<br>$136.87-154.69<br>$105.97-123.10		x	x		x	x	x	x			
47	Tchuenche (2016b)	To provide useful information on the scope of financial barriers that VMMC clients face	Follow up VMMC clients	Patient	Full economic costs, bottom up	Apr – May 2015	Average transport costs<br>$8.26 all clients<br>$9.43 urban clients<br>$7.45 peri-urban clients<br>$9.70 rural clients	N/S		x							x		Opportunity costs

IQR – Interquartile range

CI – Confidence interval

ISPA – Indirectly supervised pharmacist assistant
