# Supplementary material for: The per-patient costs of HIV services in South Africa: Systematic review and application in the South African HIV Investment Case
Source: PLoS One. 2019 Feb 26;14(2):e0210497. doi: 10.1371/journal.pone.0210497 (PMC6391029; doi:10.1371/journal.pone.0210497)
Supplement: S4 Table — (PDF) [file pone.0210497.s004.pdf]

Appendix S4: Details of ingredient-based unit costs [2017/18 ZAR]

Appendix for the paper *"The per-patient costs of HIV services in South Africa: Systematic review and application in the South African HIV Investment Case"* by Gesine Meyer-Rath, Craig van Rensburg, Calvin Chiu, Rahma Leuner and Steve Cohen

*****NB: For updates or additional details regarding unit costs based on ingredients, please contact the corresponding author at gesine@bu.edu; for updates regarding unit costs based on literature, please contact the corresponding author of the original paper.*****

1. PMTCT

**Intervention**	**PMTCT (mother not on ART, presenting in labour)**					
**Costing method**	Ingredients (NB, these are drug costs only; assumed to be incremental to standard perinatal care and postnatal visits)					
**Total cost**						**R 316.65 per mother-baby pair**
**Ingredient**	**Unit cost**	**Cost unit**	**Source of unit cost**	**Quantity**	**Source of ingredient and/ or quantity**	**Total cost**
Single dose NVP + AZT 3-hourly in labour; TDF+FTC stat	R 5.20	per child	2018 drug tender prices	1		R 5.20
NVP coverage during breastfeeding (12 months)	R 311.45	per child	2018 drug tender prices	1		R 311.45

**Intervention:**	**PMTCT (mother not on lifelong ART)**					
**Costing method:**	Ingredients (NB, these are drug costs only; assumed to be incremental to standard ante- and postnatal visits)					
**Total cost**						**R 2,125.74 per mother-baby pair**
**Ingredient**	**Unit cost**	**Cost unit**	**Source**	**Quantity**	**Source**	**Total cost**
NVP coverage during breastfeeding (6 weeks)	R 36.31	per child	2018 drug tender prices	1		R 36.31
Triple ART during pregnancy and breastfeeding	R 2,089.43	per mother	2018 drug tender prices	1		R 2,089.43

**Intervention:**	**Infant testing at birth**					
**Costing method:**	Ingredients					
**Total cost**						**R 442.23 per test**
**Ingredient**	**Unit cost**	**Cost unit**	**Source**	**Quantity**	**Source**	**Total cost**
Staff Nurse	R 2.56	per minute of staff time	2018 Government Salary Scale	20	ZoeLife	R 51.28
Counsellor	R 0.99	per minute of staff time		10	ZoeLife	R 9.92
HIV PCR	R 380.02	per test	NHLS prices March 2017	1	ZoeLife	R 380.02
Gloves	R 50.82	per pack of 50 pairs	Treasury contract RT76-2016	0.02	Own assump tion	R 1.02

Intervention:	**Infant testing at six weeks**					
**Costing method:**	Ingredients					
**Total cost**						**R 416.59 per test**
**Ingredient**	**Unit cost**	**Cost unit**	**Source**	**Quantity**	**Source**	**Total cost**
Staff Nurse	R 2.56	per minute of staff time	2018 Government Salary Scale	10	ZoeLife	R 25.64
Counsellor	R 0.99	per minute of staff time		10	ZoeLife	R 9.92
HIV PCR	R 380.02	per test	NHLS prices March 2017	1	ZoeLife	R 380.02
Gloves	R 50.82	per pack of 50 pairs	Treasury contract RT76-2016	0.02	Own assumption	R 1.02

2. Social behaviour change communication (SBCC)

Intervention	SBCC campaign 1 (message: adolescent testing, multiple partners)
**Costing method**	Expenditure (personal communication, implementing agencies)
**Total cost**	**R 12.10 per person reached**

Intervention	SBCC campaign 2 (message: condom usage)
**Costing method**	Expenditure (personal communication, implementing agencies)
**Total cost**	**R 6.19 per person reached**

Intervention	SBCC campaign 3 (message: testing, condom usage)
**Costing method**	Expenditure (personal communication, implementing agencies)
**Total cost**	**R 3.39 per person reached**

3. Comprehensive condom programming

Intervention:	**Condom distribution**					
**Costing method:**	Ingredients					
**Total cost**						**R 0.74 per condom distributed**
**Ingredient**	**Unit cost**	**Cost unit**	**Source**	**Quantity**	**Source**	**Total cost**
Male condom + distribution	R 0.63	per condom	2015-18 Condom tenders	0.98	2015-18 Condom tenders	R 0.62
Female condom + distribution	R 6.16	per condom	2015-18 Condom tenders	0.02[1]	2015-18 Condom tenders	R 0.13

[1] These represent the relative quantities of male and female condoms planned in the 2012 condom tender.

Intervention:	Male and female condom education					
**Costing method:**	Literature, updated from [1]					
**Total cost**						**R 66.06 per person trained**
**Ingredient**	**Unit cost**	**Cost unit**	**Source**	**Quantity**	**Source**	**Total cost**
Male condom	R 0.52	per condom	2015-18 Condom tenders	1	[1]	R 0.52
Female condom	R 6.14	per condom	2015-18 Condom tenders	1	[1]	R 6.14
Counsellor (first session)	R 0.99	per minute of staff time	2018 Government Salary Scale	30	[1]	R 29.70
Counsellor (follow-up session)	R 0.99			30	[1]	R 29.70

4. Care and treatment

Intervention:	ART
**Costing method:**	National ART Cost Model (NACM) 2017/18
**Total cost**	**R 3,318.62 per patient year (all adults)**<br>**R 2,760.87 per adult on first line treatment**<br>**R 2,771.13 per adult in first line treatment failure**<br>**R 8,705.15 per adult on second line ART**<br>**R 9,272.15 per adult in second line treatment failure**<br>**R 18,688.12 per adult on third line ART**<br><br>**R 3,784.19 per patient year (children)**<br>**R 3,720.73 per child on first line treatment**<br>**R 4,466.98 per child in first line treatment failure**<br>**R 4,763.82 per child on second line ART**

Intervention:	Palliative care
**Costing method:**	Personal communication, A. Lolliot
**Total cost**	**R 948.48 per patient**

Intervention:	Inpatient care
**Costing method:**	Updated from literature [2], using different CD4 strata
**Total cost**	**R 1,694.94 per patient year (pre-ART, <200 cells/microl)**<br>**R 989.47 per patient year (pre-ART, 200-349 cells/microl)**<br>**R 808.77 per patient year (pre-ART, 350-500 cells/microl)**<br>**R 393.23 per patient year (pre-ART, >500 cells/microl)**<br>**R 2,079.97 per patient year (ART, <200 cells/microl)**<br>**R 989.47 per patient year (ART, 200-349 cells/microl)**<br>**R 808.77per patient year (ART, 350-500 cells/microl)**<br>**R 393.23 per patient year (ART, >500 cells/microl)**

5. Medical male circumcision

Intervention:	Medical male circumcision
**Costing method:**	Literature [3]
**Total cost**	**R 1,770.29 per circumcision**

Intervention:	**Early infant male circumcision**
**Costing method:**	Ingredients
**Total cost**	**R 885.14 per circumcision (assumed to be 50% of adult circumcision)**

6. HIV counselling and testing (HCT)

**Intervention:**	**General population HCT (negative result)**					
**Costing method:**	Ingredients Update from literature [4]					
**Total cost**						**R 48.24 per test**
**Ingredient**	**Unit cost**	**Cost unit**	**Source**	**Quantity**	**Source**	**Total cost**
Rent and electricity	R 3,181.95	per month	Estimate	0.001	Assumed	R 3.18
Security guard	R 0.55	per minute of staff time	2018 Government Salary Scale	0.5	[9]	R 0.27
Counsellor	R 0.99	per minute of staff time	2018 Government Salary Scale	17	[9]	R 16.86
Clerk	R 1.29	per minute of staff time	2018 Government Salary Scale	4	[9]	R 5.17
Furniture and equipment	R 1 178	rental cost per month	Retail quote	0.001	Assumed. Equipment supports 1,000 patients seen per month	R 1.18
Paper flip file	R 5.00	per material	Retail quote	1	Assumed	R 5.00
Bond paper	R 58.80	per 500 pages	Retail quote	0.004	Assumed 2 sheets used	R 0.36
Biosscrub	R 155.18	Per 5L	Treasury contract	0.0002	Assumed	R 0.03
Gloves	R 50.82	per pack (100)	Treasury contract RT76-2016	0.02	RSA GF proposal for continued funding, 2013	R 1.03
Cotton wool swabs	R 81.65	per pack (100)	Treasury contract	0.01	RSA GF proposal for continued funding, 2013	R 0.82
First response test kit	R 8.17	per test	Treasury contract RT41-2017	1.01	RSA GF proposal for continued funding, 2013	R8.25
Biohazard bag	R 0.09	per bag	Treasury contract	1	RSA GF proposal for	R 0.09

					continued funding, 2013	
Result card	R 0.76	per card	Retail quote	1.01	Assumption	R 0.77
Male condom	R 0.52	per condom	Treasury contract	10.01	Assumption	R 5.23

**Intervention:**	**General population HCT (positive result)**					
**Costing method:**	Update from literature [4]					
**Total cost**	**R 74,83 per test**					
**Ingredient**	**Unit cost**	**Cost unit**	**Source**	**Quantity**	**Source**	**Total cost**
As above, plus:						
Advance test kit	R 6.50	per test	Treasury contract RT41-2017	1.01	RSA GF proposal for continued funding, 2013	R 6.57
First response test kit	R 8.17	per test	Treasury contract RT41-2017	0.007	Assumed	R 0.06
Advance test kit	R 6.50	per test	Treasury contract RT41-2017	0.007	Assumed	R 0.05
ELISA	R 55.18	per test	NHLS 2018 price list	0.0002	Assumed	R 0.01
Counsellor	R 0.99	per minute	April 2018 Government Salary Scale	20	Assumed	R 19.90

**Intervention:**	**Provider-initiated HCT**
**Costing method:**	Updated from literature [4]
**Total cost**	**R 56.07 per test (negative result)**<br>**R 82.66 per test (positive result)**

**Intervention:**	**Mobile HCT**
**Costing method:**	Updated from literature [5]
**Total cost**	**R 74.48 per test (negative result)**<br>**R 87.92 per test (positive result)**

**Intervention:**	**Home-based HCT**
**Costing method:**	Updated from literature [5]
**Total cost**	**R 69.73 per test (negative result)**<br>**R 77.42 per test (positive result)**

**Intervention:**	**HCT invitations to male pregnancy partners (negative result)**					
**Costing method:**	Ingredients					
**Total cost**	**R 50.79 per test**					
**Ingredient**	**Unit cost**	**Cost unit**	**Source**	**Quantity**	**Source**	**Total cost**
HCT invitation card	R 1.82	per card	Retail quote	1	Assumption	R 1.82
BCC material design	R 5,600.00	per day	RSA Global Fund	0.1	Assumption	R 0.56

BCC material translation	R1,238.18	per day	Retail quote	0.07	Assumption	R 0.17
General population HCT (negative)	R 48.24	per test	See above	1		R 48.24

**Intervention:**	**HCT invitations to male pregnancy partners (positive result)**					
**Costing method:**	Ingredients					
**Total cost**						**R 79.87per test**
**Ingredient**	**Unit cost**	**Cost unit**	**Source**	**Quantity**	**Source**	**Total cost**
HCT invitation card	R 1.82	per card	Retail quote	1	Assumption	R 1.82
BCC material design	R 5,600.00	per day	RSA Global Fund	0.1	Assumption	R 0.56
BCC material translation	R1,238.18	per day	Retail quote	0.07	Assumption	R 0.17
General population HCT (positive)	R 74,83	per test	See above	1		R 77.32

**Intervention:**	**Workplace HCT (negative result)**					
**Costing method:**	Ingredients					
**Total cost**						**R 55.50 per test**
**Ingredient**	**Unit cost**	**Cost unit**	**Source**	**Quantity**	**Source**	**Total cost**
Mobile van	R 2,108.26	per month		0.001	Assumption	R 2.11
Senior nurse	R 26 360	per month	2018 Government Salary Scale	0.001	Assumption	R 26.36
Community health workers (*4)	R 2,631.75	per month	Global fund proposal	0.001	Assumption	R 10.53
Consent form	R 108.00	per 500 forms	Retail quote	0,002	Assumption	R 0.22
Bioscrub	R 155.18	per 5L	Treasury contract	0.00101	Assumption	R 0.16
Gloves	R 50.82	per pack 100	Treasury contract RT76-2016	0.02	RSA GF proposal for continued funding, 2013	R 1.03
Cotton wool swabs	R 81.25	per pack 100	2015-17 NDOH bandages & dressings tender contract	0.01	RSA GF proposal for continued funding, 2013	R 0.82
First response test kit	R 8.17	per test	Treasury contract RT41-2017	1.01	RSA GF proposal for continued funding, 2013	R 8.25
Biohazard bag	R 0.09	per material	Retail quote	1.01	Assumption	R 0.09
Results card	R 0.76	per card	Retail quote	1.01	Assumption	R 0.77
Ground travel	R 3.55	per km	SARS rate	2	Assumption	R 7.10

Intervention:	Workplace HCT (positive result)					
**Costing method:**	Ingredients					
**Total cost**	**R 63.14 per test**					
**Ingredient**	**Unit cost**	**Cost unit**	**Source**	**Quantity**	**Source**	**Total cost**
As above, plus:						R 55.50
Advance test kit	R 6.50	per test	Treasury contract RT41-2017	1.01	RSA GF proposal for continued funding, 2013	R 6.57
First response test kit	R 8.17	per test	Treasury contract RT41-2017	0.06	Assumption	R 0.48
Advance test kit	R 6.50	per test	Treasury contract RT41-2017	0.06	Assumption	R 0.38
ELISA	R 55.18	per test	NHLS 2018 price list	0.004	Assumption	R 0.22

Intervention:	Testing of adolescents (negative result)					
**Costing method:**	Ingredients					
**Total cost**	**R 104.34 per test**					
**Ingredient**	**Unit cost**	**Cost unit**	**Source**	**Quantity**	**Source**	**Total cost**
Mobile van	R 2,108,26	per month	Updated based on [6]	0.0003	Assumption	R 0.59
Administrative assistant	R 19,451.74	per month	2018 Government Salary Scale	0.0003	Assumption	R 5.43
Senior nurse	R 26,359.83	per month	2018 Government Salary Scale	0.001	Assumption	R 29.43
Community health worker	R 2,631.75	per month	RSA GF proposal for continued funding, 2013	0.007	Assumption	R 17.63
Bond paper	R 58.80	per 500 pages	Retail price	0.002	Assumption	R 0.12
Data technician	R 16,319	per month	2018 Government Salary Scale	0.0001	Assumption	R 2.28
Driver	R 17,995	per month	2018 Government Salary Scale	0,001	Assumption	R 20.09
Cleaner	R 3,775	per month	2018 Government Salary Scale	0.0001	Assumption	R0.53
Bioscrub	R 155.18	per 5L	Treasury contract	0.0002	Assumption	R 1.03
First response test kit	R 8.17	per test	Treasury contract RT41-2017	1.01	Assumption	R 8.25
Biohazard bag	R 0.09	per bag	Retail quote	1.01	Assumption	R 0.09
Cotton wool swab	R 81.65	per 100	Treasury contract	0.01	Assumption	R 0.82

Male condom	R 0.52	per condom	Treasury contract	10	Assumption	R 5.18
Results card	R 0.76	per card	Retail quote	1	Assumption	R 0.76

**Intervention:**	**Testing of adolescents (positive result)**					
**Costing method:**	Ingredients					
**Total cost**						**R 111.64 per test**
**Ingredient**	**Unit cost**	**Cost unit**	**Source**	**Quantity**	**Source**	**Total cost**
As above, plus:						
Advance test kits	R 6,50	per test kit	Treasury contract RT41-2017	1.01	Assumption	R 6.57
First response test kit	R 8,17	per test kit	Treasury contract RT41-2017	0.06	Assumption	R 0.48
Advance test kits	R 6,50	per test kit	Treasury contract RT41-2017	0.006	Assumption	R 0.04
Elisa	R 55,18	per test	NHLS 2018 price list	0.004	Assumption	R 0.22

7. Other bio-medical prevention interventions

**Intervention:**	**Pre-exposure prophylaxis (example for PrEP to young women)**	
**Costing method:**	Ingredients	
**Total cost**		**R 1,917.62 per patient year (first year)**<br>**R 1,647.07 per patient year (every year thereafter)**
	**First year**	**Every year thereafter**
Drugs	R 810.84	R 810.84
Labs	R 468.63	R 226.51
HTS	R 104.21	R 100.62
STI	R 37.69	R 33.70
Social mobilisation	R 109.82	R 109.82
Counselling/ dispensing	R 138.33	R 134.35
Combination prevention	R 33.33	R 32.33
Overheads	R 149.01	R 133.12
Training	R 60.07	R 60.07
M+E	R 5.69	R 5.69

	Health system level cost	Screening & initiation	Follow-up clinical visit	Follow-up pharmacy visit	Re-initiation visit	Creatinine	Syphilis testing (follow-up)	Total cost
**Cost per visit (patient-level costs)**								
		336.43	109.46	78.60	213.30	28.71	29.72	
**Cost per visit (health-system level cost)**								
	219.11	23.55	7.66	5.50	14.93			
**Number of visits**								
First year		1	3	8	1	3	0	

Every year thereafter		-	4	8	1	1	1	

Intervention:	Post-exposure prophylaxis					
**Costing method:**	Ingredients					
**Total cost**						**R 1,093.20 per patient year**
**Ingredient**	**Unit cost**	**Cost unit**	**Source**	**Quantity**	**Source**	**Total cost**
PEP regimen (TDF, 3TC and ATV/Rit)	R 418.04	per month	2018 NDOH contract prices	1	2008 SAHIVSOC guidelines	R 418.04
Space	R 110.00	per square meter	Estimate	0.007	Assumed	R 0.73
Electricity	R1.97	per square meter	Estimate	0.007	Assumed	R 0.01
Social Worker	R 4.36	per minute of staff time	April 2018 Government Salary Scale	27.78	GF Budget 2013-2016	R 121.07
Counsellor	R 0.99	per minute of staff time	April 2018 Government Salary Scale	277.76	GF Budget 2013-2016RSA	R 275.46
Driver NGO	R 0.84	per minute of staff time	Estimate	27.78	Estimate	R 23.41
Monitoring & Evaluation officer NGO	R 2.15	per minute of staff time	RSA Global Fund Grant portfolio budgets 2018	13.89	Assumed	R 29.81
Programme Manager	R 7.02	per minute of staff time	RSA Global Fund Grant portfolio budgets 2016	6.94	GF Budget 2013-2016	R 48.75
IEC material (design, print, distribute)	R 2.47	per material	RSA GF proposal for continued funding, 2014, inflation adjusted	1	RSA GF Proposal for continued funding, 2013	R 2.47
Patient forms - printing	R 0.10	per form	RSA GF proposal, inflation adjusted	1	RSA GF Proposal for continued funding, 2013	R 0.10
HIV rapid test (average cost of all test kits)	R 6.95	per test	Treasury contract RT41-2017	1.31	Assumed (for every 100 people tested, there will be 30 retests (HIV+ve + discordant tests) and 1 kit wasted	R 9.10
Comfort packs	R 147.90	per pack	Provincial DOH CG	1	NACOSA GF Budget 2013-2016	R 147.90

			Business Plans			
Needle	R 13.11	per pack 100	Contract HM08-2015SYR	1	RSA GF Proposal for continued funding, 2013	R 0.01
Gloves	R 50.82	per pack 100	RSA GF proposal	0.01		R 1.02
Cotton wool swabs	R 81.65	per pack 100	Treasury tender contract	0.01		R 0.82
Rapid HIV test disposal	R 74.09	per 5l container	2013 RSA GF proposal, inflated	0.001		R 0.07
Phone	R 449.00	rental cost per month	Retail price	0.0064	Assumed	R 2.87
Ground travel	R 3.61	per km	SARS rate	3.2	Assumed	R 11.55

REFERENCES

1. Callegari, L., C. C. Harper, A. van der Straten, M. Kamba, T. Chipato and N. S. Padian (2008). "Consistent condom use in married Zimbabwean women after a condom intervention." Sex Transm Dis 35(6): 624-630
2. Meyer-Rath, G., A. T. Brennan, M. P. Fox, T. Modisenyane, N. Tshabangu, L. Mohapi, S. Rosen and N. Martinson (2013). "Rates and cost of hospitalization before and after initiation of antiretroviral therapy in urban and rural settings in South Africa." J Acquir Immune Defic Syndr 62(3): 322-328.
3. Tchuenche M, Haté V, McPherson D, Palmer E, Thambinayagam A, Loykissoonlal D, et al. Estimating client out-of-pocket costs for accessing voluntary medical male circumcision in South Africa. PLoS One. 2016;11(10):1–10.
4. Bassett, I. V., D. Govindasamy, A. S. Erlwanger, E. P. Hyle, K. Kranzer, N. van Schaik, F. Noubary, A. D. Paltiel, R. Wood, R. P. Walensky, E. Losina, L. G. Bekker and K. A. Freedberg (2014). "Mobile HIV screening in Cape Town, South Africa: clinical impact, cost and cost-effectiveness." PLoS One 9(1): e85197.
5. Smith, J. A., M. Sharma, C. Levin, J. M. Baeten, H. van Rooyen, C. Celum, T. B. Hallett and R. V. Barnabas (2015). "Cost-effectiveness of community-based strategies to strengthen the continuum of HIV care in rural South Africa: a health economic modelling analysis." Lancet HIV 2(4): e159-e168.
6. Tabana H, Nkonki L, Hongoro C, Doherty T, Ekström AM, Naik R, et al. (2015) A Cost-Effectiveness Analysis of a Home-Based HIV Counselling and Testing Intervention versus the Standard (Facility Based) HIV Testing Strategy in Rural South Africa. PLoS ONE 10(8): e0135048. doi:10.1371/journal.pone.0135048
